# To wear or not to wear? Unpacking the #NoMask discourses and conversations on Twitter

**DOI:** 10.1007/s43545-022-00556-9

**Published:** 2022-11-21

**Authors:** Anita Lavorgna, Les Carr, Ashton Kingdon

**Affiliations:** 1 Department of Sociology, Social Policy and Criminology, University of Southampton, Southampton, UK; 2 Web Science Institute, University of Southampton, Southampton, UK; 3 Department of Political and Social Sciences, University of Bologna, Southampton, UK

**Keywords:** COVID-19, Masks, Medical misinformation, Social media analysis, Twitter

## Abstract

In the context of the COVID-19 health crisis, the use of face masks has been a topic broadly debated. In many Western countries, especially at the heights of the pandemic, discussions on the use of protective facemasks were often linked to what were mainly political considerations, often fueled by health-related misinformation. Our study brings together social sciences and computer science expertise to retrospectively unpack the #NoMask discourses and conversations using both network analysis approaches on big data retrieved from Twitter and qualitative analyses on sub-sets of relevant social media data. By looking comparatively at two dataset gathered at different stages of the health crisis (2020 and 2022), we aim to better understand the role of Twitter in that interesting area where the dissemination of health misinformation became capitalized by the political narrative linking the social discontent caused by the socio-economic impacts of the pandemic to specific political ideologies. Our analyses show that there has never been a unique ‘NoMask movement,’ nor a defined online community. Rather, we can identify a range of relatively niche, loosely connected, and heterogeneous actors that, in the course of the pandemic, independently pushed diverse (but converging and compatible) discourses. Conversations directly linked to the #NoMask relevant hashtags are overall limited, as twitters using them are not talking to each other; nonetheless, they successfully engaged a larger audience.

## Introduction

Since the beginning of the COVID-19 health crisis, wearing (or not wearing) a facemask has been a hot topic. The presence of conflicting advices in the earliest stages of the pandemic—when, for instance, the World Health Organization (WHO) itself was recommending not to wear masks if you were not sick or caring for someone who was sick (CNN 2020)—has likely contributed to the spread of polluted and misleading information on the topic. Over time, however, the importance to use masks became supported by a growing number of scientific studies; even if most masks do not provide full protection, they are critical tools in curbing the spread of the virus. In light of new scientific evidence, the WHO itself changed its recommendations (WHO 2020).

Since the early stages of the pandemic, some political groups have exploited the situation, backing the politicization of masks as a way to minimize the impact of the pandemic in the effort to preserve the economy and please a part of the electorate or as a pushback against what they viewed as government overreach (e.g., Il Post 2020; Independent 2020; Politico 2020a; The Washington Post 2020). Problematically, in this scenario health-related misinformation has been spread to back-up what are mainly political considerations. Because of their features and algorithmic architecture, social media had a major role in this context, facilitating the promotion of political agendas through the dissemination of health misinformation and enabling the capitalization of the political narrative linking the discontent caused by the socio-economic impacts of the pandemic to specific political ideologies, in line with recently published literature carried out in the context of the pandemic looking, among other things, at partisan motivated reasoning and selective sharing (Barbieri and Bonini 2021; Freiling et al. 2021; Lavorgna 2021; Rooke 2021). The rise of a ‘no-mask’ or ‘anti-mask’ movement in many Western countries has been reported, and compared with the anti-vax movement as activists became a vocal force in opposing important preventative efforts for public health (Politico 2020b). Indeed, this is not surprising as aligned with findings from previous studies on misinformation, which have shown how this latter significantly impacts the social and economic fabric of societies, as well as public health, by reinforcing people’s opinion by repeated exposure to false, inaccurate, or malicious information (for a meta-analysis, see Ha et al. 2021).

In our exploratory study, which brings together sociological and computer science expertise, we aim to retrospectively unpack the #NoMask conversations that occurred in 2020 and at the beginning of 2022 using both network analysis approaches on big data retrieved from Twitter, and qualitative analyses on sub-sets of relevant social media data in order to (1) explore the most prominent themes associated with the #NoMask discourses and (2) study the interactions between relevant accounts. Our analyses show that there has never been a unique ‘NoMask movement’ nor a defined online community. Rather, we can identify a range of relatively niche, loosely connected, and heterogeneous actors who independently pushed diverse (but converging and compatible) discourses. Conversations directly linked to the #NoMask relevant hashtags are overall limited, as users using them are not talking to each other; nonetheless, they successfully engaged a larger audience.

## Background

### Polarization and politicization of preventive measures

In the unfolding of the COVID-19 pandemic, we have witnessed stormy debates in many countries, opposing views pushing for more stringent preventive measures to views promoting ‘business as usual.’ The stances at the core of these debates have been at times different depending on the specific country and shifting in the course of the pandemic also with *U* turns depending on the changing infection rates. Overall, however, a polarized divide between risk-prone and risk-averse groups with regards to preventive measures can be identified throughout in public debates online—which is not surprising, as researchers have long stressed the polarized nature of health-related online debates on (perceived) controversial topics (Gunnarsson Lorentzen 2014; Di Ronco and Allen-Robertson 2019).

This polarization, at least in the Anglosphere and in other European countries the authors are familiar with, seems to be aligned with diverging political views (in line with what has been recently observed in Lavorgna and Carr 2021). Recent studies and commentaries on the pandemic have suggested how, besides being a public health crisis, the Coronavirus pandemic has been also a political communication and health communication crisis (Gollust et al. 2020; Kreps and Kriner 2020; Lovari 2020; Barbieri and Bonini 2021; Bucchi 2021; Lavorgna 2021). Boberg and colleagues (2020), discussing the ‘pandemic populism’ surrounding the health crisis, emphasized how some media outlets, shared through social media, contributed to further societal confusion, and the spreading of politicized messages during the pandemic; social media, indeed, became a catalyst for the politicization of the pandemic (Yang et al. 2020).

The politicization of what should be factual, scientific information is very problematic, as this can lead to long-term problems, fueling science skepticism among citizens and scientists themselves (Druckman 2017), and fostering the spread of misinformation (Righetti 2020). From a psychology of misinformation perspective, we know that partisanship affects information processing and that misinformation is persistent, difficult to erase from people’s memory (Lewandowsky 2020; Vegetti and Mancosu 2020). It should not be forgotten that, in our ‘post-truth’ political arena, misinformation is often disseminated by political actors for a specific purpose, being considered a rational strategy to be used in pursuit of political objectives, rather than a coincidental by-product of some other activity (Lewandowsky 2020). Hence, it is relevant that Yang and colleagues (2020), analyzing the spreading of low-credibility information on Twitter during the pandemic, found the presence—alongside likely humans, counting for the majority of volume of tweets—of social bots involved in both posting and amplifying misinformation in a coordinated fashion.

The success of misinformation, however, generally depends of the existence of a context where fact-checking and evidence-based reasoning do not always rule, experts and expertise are dismissed, and populist politics is on the rise (Nguyen and Catalan-Matamoros 2020). From this perspective, the recent development of the online #NoMask (or #AntiMask) diatribes during the pandemic can be viewed the latest manifestations of uncivil online discussions and the spread of polluted information that started way back (Nguyen and Catalan-Matamoros 2020) and that resurface every time that (apparently) controversial areas of health and science become the flavor of the month.

### #NoMask propaganda and misinformation campaigns

Some groups that have been reportedly very active in spreading misinformation during the pandemic are fringe groups supporting and propagating conspiratorial thinking (Forbes 2020; The Guardian 2020). This is not necessarily surprising considering that, beyond the immediate public health emergency, the global pandemic-led crisis has had profound effects on governance, social polarization, and political and ideological discourses, all of which greatly influence the ways in which certain beliefs and being packaged and disseminated (Christou 2020; Eger 2020; Silke 2020; Wodak 2020). Not since the attacks on September 11, 2001 has a global event provided so much opportunity to conspiracy theorists, with the fear, panic, uncertainty, and rapidly climbing death rates resulting from the Coronavirus creating the ideal atmosphere for the flourishing of alternative explanations regarding its origins (Cosentino 2020; Gollust et al. 2020). With global lockdown measures constraining millions within their homes, the opportunities for extremists to utilize technology to radicalize, recruit, and disseminate propaganda has grown exponentially (Christou 2020; Daymon 2020). In addition to such extremist propaganda, the pandemic has seen deliberate and concentrated disinformation campaigns, with the environment of fear and chaos being exploited by malicious actors to spread false information and conspiracy theories (Argentino 2020; Tobi 2021).

Some of the most prominent conspiracy theories relating to COVID-19 are intertwined with the so-called QAnon (‘*Q*’) phenomenon (see also Amarasingam and Argentino 2020; Consentino 2020; McManus et al. 2020; The Atlantic 2020; Tabatabai 2020). QAnon represents the first ‘born digital’ meta-conspiracy theory representing a militant anti-establishment ideology rooted in an apocalyptic desire to destroy the existing corrupt world and its growth due to the influence of social media and the effects of the COVID-19 pandemic has been unprecedented. Following the Coronavirus outbreak in January 2020, followers of Q have downplayed the severity of the crisis and amplified medical disinformation: for instance, research from Argentino (2020) highlighted that QAnon influencers in early 2020 followed the lead of Trump in downplaying the threat of the virus, referring to is as a ‘hoax’ and maintaining it was part of a plot to damage Trump’s chances of re-election.

Conspiracy theorists and peddlers of alternative health facts have been circulating various disinformation campaigns via social media regarding specifically the negative impacts of wearing a mask; in the course of 2020, these were particularly prominent on Far Right and QAnon Telegram channels (Argentino 2020; BBC 2020). Some groups on Facebook, then removed from the platform, even disseminated printable ‘Mask Exemption Cards’ issued by the ‘Freedom to Breath’ agency, encouraging group members to show them to authorities as a way of protesting mask requirements (The Verge 2020).

In the pandemic context and through polarized online discussions, hence, for some the mask became a symbol through which people can create, use, and distribute a certain social identity, coming to represent who we believe we are and how we want the others to see us (Copes and Ragland 2016; Copes et al. 2019; Lavorgna 2021). After all, anomalous objects and bodies often come to be especially important to driving social and institutional dynamics—either as totemic objects which form a basis of unity and cohesion or as objects of contention, dispute, or individualization (Brown 2020).

### #NoMask in the context of the ‘hashtag movements’

Social media platforms, and the popular microblogging service Twitter among those, have received increased scholarly attention during the past decade (e.g., Murthy 2012). Twitter in particular has been explored and analyzed as a method for circulating information during a disaster, a way for companies to market their services, a space for community building, and as a site of activist gestures (as summarized by Dadas 2017). For this latter purpose, the uses of hashtags in particular have been studied, to the point that the term ‘hashtag activism’ has been coined to describe ‘when large numbers of postings appear on social media under a common hashtagged word, phrase, or sentence with a social or political claim,’ giving them ‘a narrative form and agency’ (Yang 2016: 13). Hashtags, indeed, are nothing else than a form of digital ‘prosumption’ producing a fundamental way in which content is organized, accessed, and circulated and vital metadata for researchers (Lupton 2014). In this context, Twitter has been interpreted as ‘a site of resistance,’ prompting individuals from within and outside a certain community to recognize issues and respond to them (Williams 2015: 344); and ‘a central component in social movement building’ (Storer and Rodriguez 2020: 160).

Some studies have been documenting how elements of Twitter have been used to form online communities or even to enhance the engagement and visibility of certain subcultures (e.g., Gruzt et al. 2011; Wignall 2017; de Saint Laurent 2020). Because of the way Twitter is structured (e.g., its emphasis on concision, the retweet function), tweets and their hashtags have the potential for a very broad reach and to encourage the rapid spread of information beyond the author’s visible network, gathering quickly attention to a cause (Dadas 2017)—sometimes globally, even if contextual realities, institutional norms and local politics can constrain and (re)frame the issues discussed in the Twittersphere (Ofori-Parku and Moscato 2018). Consider, for instance, the notorious case of the #MeToo movement and its transnational dimension (Xiong et al. 2019; Lee and Murdie 2020).

The deliberate use of #NoMask in online setting, therefore, could be considered as an attempt to use social media and social networking sites as an act of activism and resistance, at least in the eyes of some of those embracing the use of this hashtag. While we recognize this possibility and that similarly to studies focusing on hashtag movements we aim to better understand the role of Twitter in in-group identities, our focus in this contribution is rather in unpacking the ways in which the dissemination of health misinformation becomes capitalized by the political narrative linking the social discontent caused by the socio-economic impacts of the pandemic to specific political ideologies. Our core research questions are as follows: what are the main themes receiving attention in the Twittersphere in association with #NoMask discourses? And how, and to what extent, are the accounts engaging in these discourses interacting among each other?

## Methodology

**Fig. 1:**
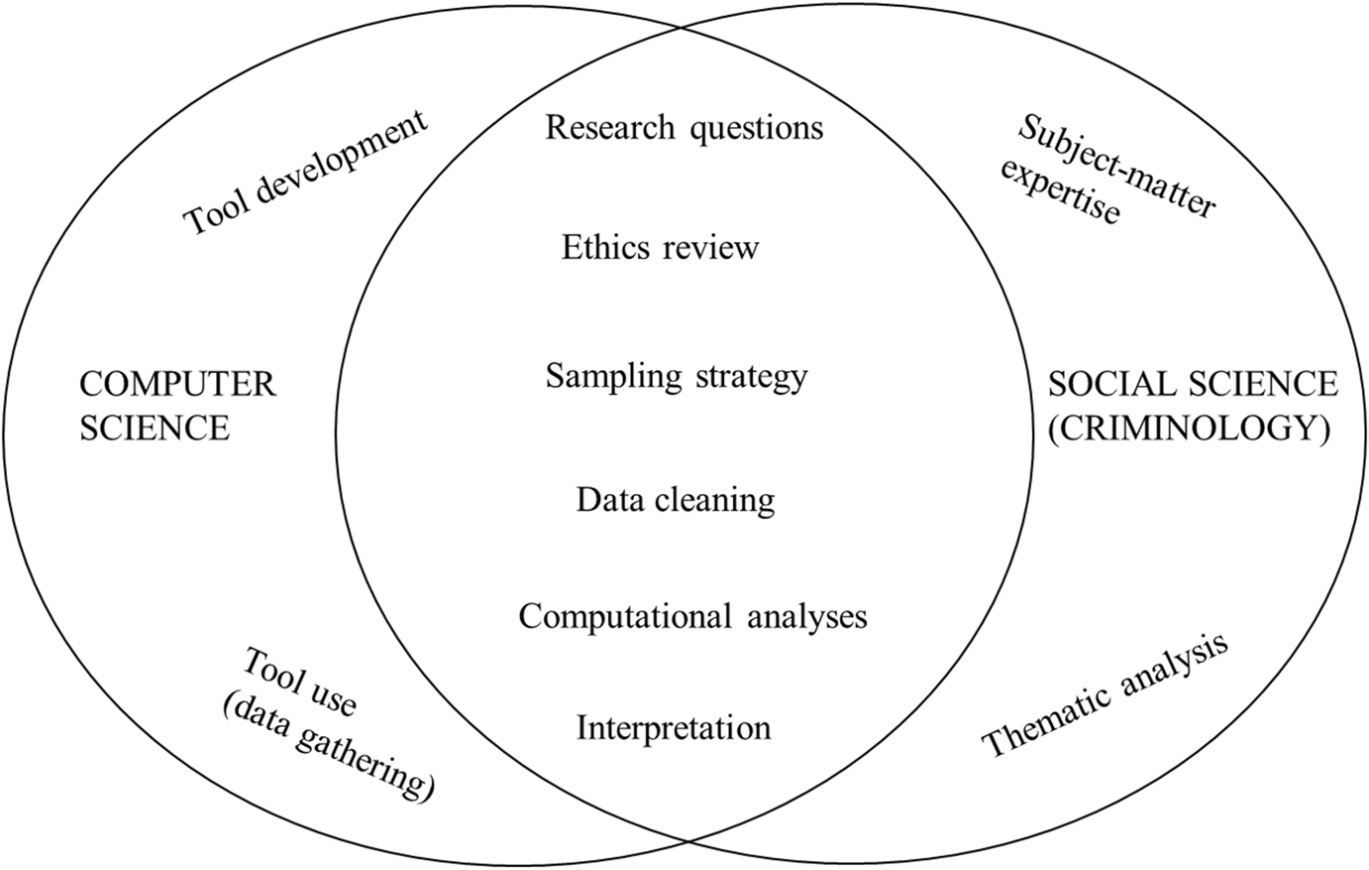
The socio-technical approach used

This study has been carried out collaboratively by researchers in the social and computer sciences through an exploratory and iterative process with the goal to use both computational power and qualitative approaches to untangle our research puzzle in a more comprehensive way. The socio-technical approach adopted promoted a constant interaction between the computer scientist managing the data collection and network analyses and the social scientists providing subject matter expertise and enabled us to observe general trends and well as to ‘zoom in’ and analyze more in-depth sub-sets of data of particular relevance, as summarized in the following Fig. 1.

### Data collection

Tweets were collected in two separate stages: (1) for a five-month period of observation from 1st February 2020 until 30th June 2020^1^; (2) for two additional months from January 1st 2022 to February 28th 2022^2^ Tweets were collected if they included any of the four key hashtags #NoMasks, #NoMask, #antimasks, or #antimask which had been chosen after preliminary manual searches to determine which hashtags were more used in this context. We purposely decided to focus on hashtags to focus on people ‘signaling’ to others their link to those hashtags and to what they (could) represent. Indeed, hashtags are no longer only information-locating resources, but have become interpersonal resources for—among other things—coordinating social relationships and expressing affinity and affiliation (Zappavigna 2018). Additionally, capturing hashtags is a common, practical approach to capture social media conversations (Lorentzen and Nolin 2017).

We identified relevant tweets post hoc from searches in the Twitter Web app (https://twitter.com/search-advanced) using the Web Data Research Assistant software developed by one of the authors.^3^ The software uses a web browser extension to save Twitter search results into spreadsheet format for flexible collection and analysis by non-programmers. The data were retrospectively collected on 5th–6th July 2020 and on 7th–8th March 2022, hence any likes, retweets, replies, or deletions subsequent to these census periods were not included in the data. This search produced a total number of 23,645 tweets (including a total of 9856 hashtags) from 10,967 Twitter accounts over the initial 5 months considered and of 5560 tweets (with a total of 4737 hashtags) from 3300 accounts over the last 2 months considered. Further information not present on the search page (such as account profiles and follower numbers) was subsequently obtained directly from the Twitter public API.^4^

This hashtag-based collection is a subset of the larger Twitter conversation around the use of masks. A test showed that 6373 tweets using both the words ‘wear’ and ‘mask’ were posted over just a 5-h period on 10th June 2020; of these tweets only 10% used any hashtags at all and only 0.1% used the #NoMask(s) hashtags. Our decision to sample using the hashtag was made in accordance with our chosen research question to identify those tweets (and those accounts) that deliberately and explicitly align with the public discourses on Twitter on our topic of interest.

Of course, relying on Twitter data has several limitations, especially as regards potential demographic biases, issues of social media access, and unknown provenance of some data (as discussed, e.g., in Blank 2017; Halford et al. 2018). Also, relevant conversations were played out in more social platforms than Twitter and in more spaces than simply online. Nonetheless, Twitter allowed us to examine some aspects of interest of these wider conversational engagements. An additional limitation that needs to be recognized is that our study, as a result of the keyword used, focuses on a Western, northern-centric basis and has an English-speaking bias. Further research could test our approach and findings by looking at different regions of the world, where misinformation could potentially follow different dynamics because of a different sociocultural substratum or a different social media usage.

In order to carry out our study we took all the necessary precautions to ensure that our research was ethically informed (Zimmer and Kinder-Kurlanda 2017): we collected data using a software tool compatible with Twitter policies, and we collected information that had already been posted on Twitter in line with its Terms and Conditions; for the nature of the information posted and the platform used we could assume that the participants expected the virtual space used to be public. For concerns related to users’ anonymity, we did not use personal identifiers. Similarly, in the results presented below we will not quote directly the content of the tweets, in line with the current best practices for digital research (e.g., Williams et al. 2017; Social Data Lab 2019). Our study plans were approved by our university Research Ethics Committee (ERGO2 55,870 and 55,870.A1).

### Data analysis

#### Stage I: reflexive thematic analysis based on purposively sampled hashtags and tweets

The first stage of analysis, based on purposive (judgmental) sampling, aimed to explore the most prominent themes associated with the #NoMask discourses at the heights of the pandemic and focused on the most used hashtags and the tweets that elicited the biggest response from other Twitter users in terms of likes, retweets, and responses. A standard nonprobability sampling technique in qualitative research, purposive sampling has well-known limitations, but was appropriate to allow us to focus on the conversations which attracted more attention (e.g., Uprichard 2013; Vehovar et al. 2016); indeed, our aim was not to capture everything, but to interpret dominant themes and trends.

Out of a total of 9856 hashtags, only 2849 were used more than once and only 462 more than ten times. Among these, only 45 were used over 100 times, and we considered only this subset (*subset Ia*) for our thematic analysis. Out of 23,645 tweets, only 149 received more than 100 likes and only 79 more than 100 retweets. By joining these selections, 76 duplicates were found, and 152 unique values remained, which we defined as the most ‘popular’ tweets. We were also interested in looking at the tweets which prompted more conversations, which could be operationalized by looking at the number of replies. Out of the full dataset of tweets, only 4558 received at least 1 reply. We defined as ‘successful’ in prompting a conversation those tweets with more of 50 replies, for a subset of 45 tweets (ranging from 50 to 618 replies each). When comparing the dataset of the ‘popular’ tweets with this latter one, we noticed that 42 duplicates were present. Considering the major overlap within these groups, we decided to join the sub-datasets for the analysis, which resulted in a final dataset (*subset IIa*) with 155 unique tweets (sent by 108 unique accounts). All relevant data from these two sub-sets were thematically analyzed manually and individually by the social science researchers. Researchers’ notes were then shared and discussed and integrated into a coherent analysis by drawing on reflexive thematic analysis (Braun and Clarke 2019).

A similar thematic analysis was carried out with the more recent dataset that encompasses data collected in a moment when many Western countries were starting to discuss the end or at least an ease of the pandemic restrictions (CNN 2022). In this second case, out of a total of 4734 hashtags, only 1362 were used more than once and only 228 more than 10 times. Among these, only 28 were used over 100 times, and we considered only this subset (*subset Ib*) for our thematic analysis. Out of 5560 tweets, only 40 received more than 100 likes and only 10 more than 100 retweets. By joining these selections, 9 duplicates were found and 41 unique values remained (the most ‘popular’ tweets for the second time period). Out of the full dataset of tweets, only 1170 received at least 1 reply and only 7 more than 50 replies (again, what we defined as ‘successful’—ranging from 51 to 855 replies each). Joining the ‘popular’ and the ‘successful’ datasets and eliminating the duplicates, the final dataset (*subset IIb*) comprised 41 unique tweets (sent by 39 unique accounts).

#### Stage II: network analysis

A second stage of analysis focused on the #NoMask conversations, looking at the interaction between the Twitter accounts (nodes in the graph visualization) that appeared in our full dataset according to four criteria (getting more focused on the conversational activity at each step):

1. The complete mention network (*N1* = 21,205; *N2* = 6843) composed of everyone who tweeted or who was mentioned in one of those tweets (even if they did not tweet using one of the hashtags under consideration themselves);
2. The basic tweet network comprising all those who actively contributed one or more tweets in our dataset (*N1* = 10,967; *N2* = 3300);
3. The internal conversational network comprising accounts in the basic Twitter network who mentioned each other or replied to each other’s tweets (*N1* = 144; *N2* = 94);
4. The successful tweet network comprising those who contributed one or more tweets that passed the threshold for significant response (*N1* = 108; *N2* = 39).

Online, the traditional toolkit of network analysis, nowadays broadly used across the social sciences, is becoming prominent, offering important opportunities to deal with the volume, variety, and velocity of digital data (see Pavan 2021). As digital media impact our personal and collective social milieus (Freeman 2002; Marres 2017), both online social and semantic networks can help us—respectively—to improve our understanding of how individual and organizational actors are tied (Wasserman and Faust 1994) and of how they produce and circulate content and, as such, represent knowledge (Diesner and Carley 2011; Pavan 2021). In our analysis, by looking at different types and aspects of relevant networks, we focus on various structural features (e.g., of the network as a whole, of prominent actors, of clusters), in line with existing literature (Newman 2018; Yousefi Nooraie et al. 2020).

Before moving to the presentation of our results, it should be stressed that the ‘NoMask’ phenomenon is not prevalent in the Twittersphere and that—at least around the time we completed our data first collection—the use of the anti-mask hashtags was limited when compared to the broader discussion about masks. To contextualize our study, it is worth to report a small ‘experiment’ we did: we used the Web Data Research Assistant to capture tweets that contained the words ‘wear’ and ‘mask’ (*N* = 6373, collected from five hours on June 10th 2020). Of these tweets, there were only four occurrences of the #NoMask hashtag. In a sample of 100 tweets, 77 were pro-mask (e.g., ‘Wear a damn mask’), 12 were anti-mask (e.g., ‘Imagine working outside still having to wear a mask’), and 11 were ambiguous (e.g., ‘You can tell he doesn’t wear his mask’). This suggests that (at least at that time) there was a much bigger Twitter conversation about masks that would appear to be strongly in favor of mask wearing.

However, we have also seen that, even if conversations occurring under the #NoMask-adjacent hashtags are overall limited, as tweeters using relevant hashtags are not talking to each other, they are relatively successful in engaging with a larger audience. This feature entails that tweeters promoting anti-mask views (and their converging and compatible narratives) are well positioned to increasingly engage with and potentially attract, users on Twitter, who might be driven toward the anti-mask narrative by one of its neighboring discourses, and vice versa.

## Results and discussion

### Heterogeneous categories and the transversal theme of freedom

By looking qualitatively at the most popular hashtags and tweets, we were able to examine in detail the main themes receiving attention in the Twittersphere in association with #NoMask discourses. First of all, the 45 hashtags comprising our *sub-dataset Ia* were manually sorted out (checking the content of the associated tweets to clarify the context) according to the following main categories: hashtags specifically relating to the unwillingness to wear masks (*N* = 9); anti-lockdown (*N* = 9); pro-Trump (*N* = 7); neutral/informative (*N* = 6); anti-vaxxers (*N* = 5); conspiracy theories linked to QAnon (*N* = 4); anti-government movements, including extremist groups^5^ (*N* = 3); and pro-mask (*N* = 2). This first stage of analysis immediately confirmed the presence of QAnon promoters and other politically-oriented movements in the dominant anti-mask discourses, together with anti-lockdown supporters and anti-vaxxers, and made clear how the discourses were all predominantly US-focused (for instance, none of the top hashtags referred explicitly to the politics of other countries, including other Anglophone countries). The prevalence of US-focused discourses could be linked to the fact that the USA have, by far, the larger number of Twitter users (Statista 2021). Additionally, it has to be considered that the timeframe analyzed was ahead of the 2020 US Presidential election; in the first half of 2020 public debates were already heated as the Democratic Party presidential primaries and caucuses were held.

Similarly, the 28 hashtags comprising our *sub-dataset Ib* were sorted according to the following main categories: hashtags specifically relating to anti-vaxxers (*N* = 14, including references to the ‘Freedom Convoy 2022^6^’)^7^; the unwillingness to wear masks (*N* = 10, with a stronger focus, compared to the first dataset, on the opposition to the use of masks for children, and freedom rights in general); anti-restrictions (in general) (*N* = 3); and attacking Biden politically (*N* = 1). Again, most discourses were US-focused, with a clear political undertone. Not surprisingly, because of the slackening of the pandemic restrictions and changes in the (US) domestic policy, the focus on lockdowns and QAnon/Trump was replaced by a bigger focus on vaccination mandates.^8^

Second, we looked at the sub-sets of the most popular/successful tweets as defined above (155 in *IIa* and 41 in *IIb*). Also in this case, we first categorized the tweets into thematic categories, depending on the main focus of their content. Some tweets were included in more than one group. Tweets were predominantly in English with the exception of 17 tweets in Italian and one tweet in Urdu in *IIa* (Table 1).

**Table 1:** Categories emerging from the most popular/successful tweets

Main categories	Number of tweets *(IIa)*	Number of tweets *(IIb)*
Straightforwardly anti-mask	115	24 (mostly welcoming the end of masks mandates or criticizing people still wearing/defending mask use. 7 tweets had a specific focus on the use of masks in school)
Pro-Trump	61 (supporting explicitly Trump 50; being negative against Fauci 6; being negative against the Democrats 5)	1
Discussing health-related issues	36 (health concerns about wearing a mask 16; discussing the health benefits of wearing a mask 7; worried about children wearing masks in school 7; health-related conspiratorial thinking 6)	1 (‘I trust my immune system’)
Conspiracy theories	44 (specifically referring to QAnon 15)	2 (general, no reference to a specific ‘theory’)
Pro-mask	21	2
Anti-lockdown/ vs social control	17	6
Anti-government movements	14	0
Other	16 (Black Lives Matter 8; promoting exemption cards 4; neutral 1; non-relevant 3)	5 (I do not have fear 1; non-relevant 4)

First of all, these categories clarify that there has never been such a thing as a unique #NoMask movement. Rather, we had several heterogeneous actors—some acting in their individual capacity, others as part of a specific movement or organization—that independently pushed, since the beginning of the pandemic, a certain narrative in line with their own personal systems of beliefs, or to promote/sustain a certain (political) agenda. For instance, it clearly emerges how, at least in the USA, since early 2020 the Coronavirus pandemic has supplied fertile ground for anti-government movements, radical right groups, and conspiracy theorists who have somehow joined forces in anti-containment protests across the nation; a similar pattern has been observed also in Italy (Lavorgna 2021).

The themes raised by these various voices are, not surprisingly, quite diverse, ranging from election politics to social control, from conspiratorial thinking to issues of race, especially during the first stages of the pandemic. In the tweets from 2022, there seems to be more homogeneity of themes, even if their focus varies (e.g., from the use of masks in schools to their use in shops, from the association of face masks with fear to the lack of trust in ‘official media’). There is, throughout, a key transversal theme that is at the basis of putting together under the same ‘hashtag umbrella’ very diverse groups, in a logic of ‘us vs them’: freedom vs tyranny (of the government, of the Deep State, of ‘official’ scientific experts and the WHO, of media). Masks, similarly to lockdowns and vaccinations, are here seen as an undue interference impacting individual and group liberties; official health recommendations are disregarded and even challenged. This is in line with the psychological mechanisms of reactance and cognitive dissonance in the context of science denial (Rosenberg and Siegel 2018; Prot and Anderson 2019): according to these mechanisms, people tend to reject scientific evidence when they perceive it as a threat to their ability to act in a certain way, and, when informed of the risks related to a particular behavior, they reduce the dissonance between their cognitions and intended behaviors by challenging the validity of the information or underestimating the risks involved with the behavior, rather than adapting their behavior. Seeking strength in numbers, individuals moving under the #NoMask-adjacent hashtags, in a way, were trying to re-establish a sense of control, after having experienced a loss of agency in the pandemic crisis; in this context, movements embracing this inclination can exploit the situation to present themselves as sympathetic allies.

Interestingly, health-related themes were overall relatively limited: only one of the dominant hashtags (*Ia*) was directly linked to questioning the effectiveness of masks from a health-related perspective (#MasksDoNotWork) and a couple more were denying the presence of a health risk, calling the pandemic a ‘hoax’ or a ‘fake.’ From the second data collection (*Ib*), while no hashtag was directly linked to health matters, some associated tweets questioned the fact that masks (or vaccines/boosters) had any proven effect. Also when we look at the tweets’ dataset, the mention of health-related issues is outnumbered by political or oppositional discourses. This finding confirms the idea that, in the context of the pandemic, at least in predominant Twitter discourses the mask has become a symbol for socio-political identification (in line with Sanders et al. 2021) and is aligned with the existing literature demonstrating political bias in the perception of what is ‘a fake news’ (e.g., van der Linden et al. 2020).

It can here be useful to open a brief parenthesis on how the concept of ‘risk’ is constructed and manifested, as we as we cannot ignore the different moral basis used to identify what a risk is in public health interventions and their supporters (i.e., through the identification of identifiable harms and victims) and by the users behind the tweets analyzed (for which, the real risk is the alleged thread to their individual freedoms) (in line with Hunt 1999; Lowe 2019). For these latter, by reconceptualizing risk as danger rather than a balance of possibilities (see Fox 1999; Lee 2014), the COVID-19-related health risk would be nothing else than a way to justify surveillance and restrictions toward the population. As such, while the concept of risk has been utilized in this study as prevalent in the relevant literature, we should recognize that—as observed in Lowe (2019)—the adoption of risk-based narratives can contain unresolved contradictions (shifting also the implicit burden of the related responsibility to manage that risk, e.g., from the uncompliant individual to the dystopic State) and consequently can be problematic in the context of public communication.

From this first stage of analysis, it also emerged how the voices of (pro-mask) opponents directly engaging in the prevalent anti-mask discourses are very limited and hence the discourses they tried to promote ended up being outnumbered. This finding resonates with the findings of Johnson and colleagues (2020) who, looking at the online diatribe surrounding vaccinations, showed how anti-vax clusters online overall provide a larger number of sites for engagement than the pro-vaccination population and offer a variety of themes and narratives, hence being able to attract the interest of a diverse population of individuals; pro-vaccination discourses, on the other hand, tend to be more isolated. In other words, pro-mask discourses (or, at least, how they are ‘pushed through’ on Twitter, especially through the use of hashtags) end up being very marginal in the overall anti-mask context. Furthermore, the tweets associated with the prominent pro-mask hashtag identified in the first dataset collected were clearly not looking for genuine dialogue with their counterparts; rather, they were openly antagonistic (for instance, referring to anti-mask users as ‘covidiots’. Similarly, the pro-mask tweets we identified (again, mostly in the first dataset collected) were also oppositional: they did not engage with any of the main themes raised by those opposing the use of masks, but they rather insisted on the stupidity (‘lack of brain’) of those opposing the use of masks and their dangerousness in terms of public health; also, they were generally referring to the ‘no-masks’ as if there a unique movement failing to recognize the multifaceted nature of voices behind the #NoMask discourses. As emerges from the analysis of *IIb*, pro-mask voices remained extremely marginal in these discourses, failing to pierce the beliefs of their counterparts or even to create a constructive dialogue with them.

### Interactions between accounts

**Fig. 2:**
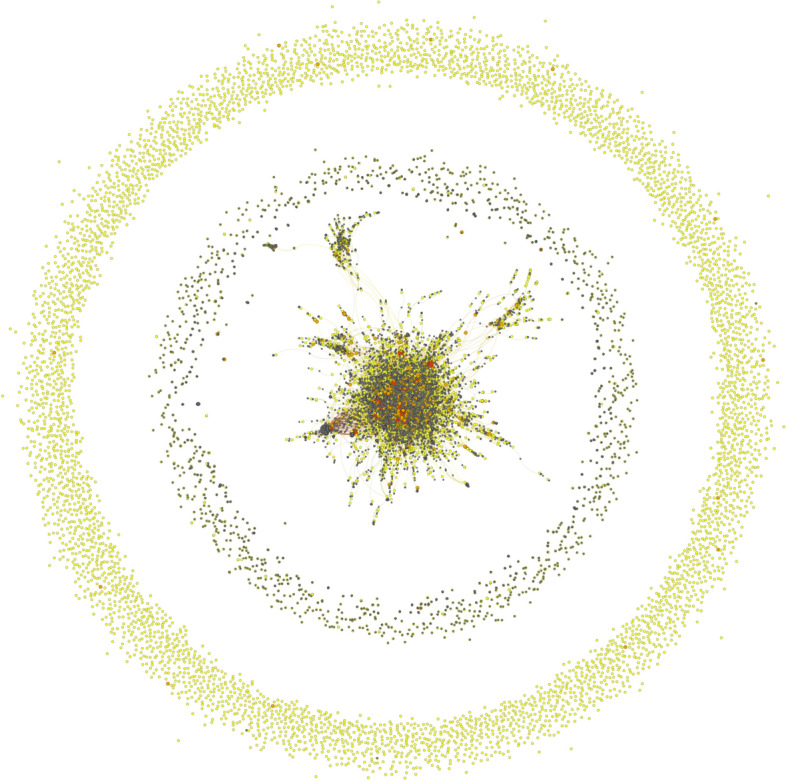
Complete mention networks (**a**). Please note: in gray are the accounts with mentions only. Darker colored nodes indicate successively more tweets contributing to the dataset or mentions: light yellow (a few); orange (tens of); and red (more than a hundred). Larger nodes are accounts with more #NoMask tweets or mentions

**Fig. 3:**
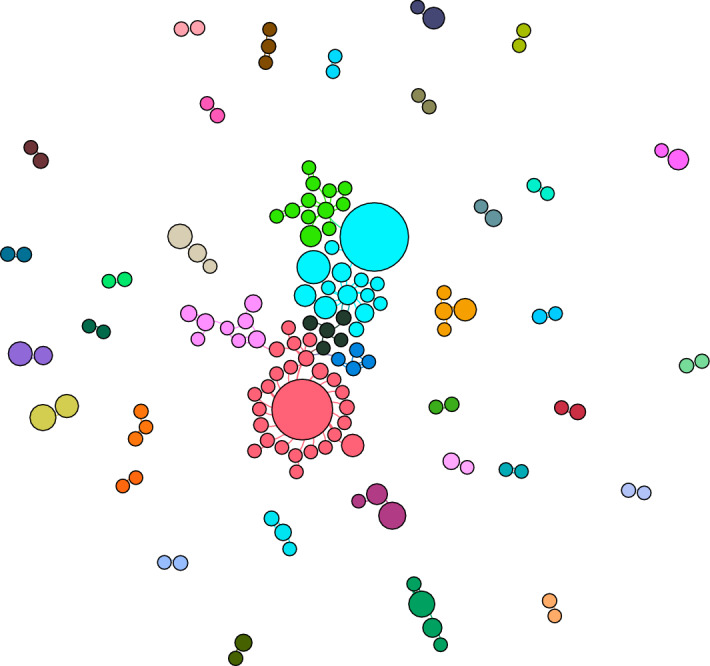
Internal conversational network (**a**). Please note: the colors show the partitioning of the network into different regions according to modularity (the internal interaction within a region over its external interaction with the rest of the network)

We initially investigated the use of the hashtags under consideration from the oldest dataset by looking both at the complete mention network [*N* = 21,205, see Fig. 2: Complete mention network (a)] and at our basic tweet network (*N* = 10,967) by scrutinizing the users who wrote those tweets in 2020 and the relationships between them to determine whether there was an obviously coordinated activity, whether there were clear relationships between the users, whether they communicated with each other, or whether they were aware of each other’s existence. In other words, we tried to determine whether our 2020 dataset was the result of a ‘community’ of anti-mask users or a set of largely independent Twitter accounts. Our results showed that although the tweeters were heterogeneous actors independently pushing diverse (but converging and compatible) discourses (they were politically MAGA Trump supporters plus a few Italian sovereigntists, free-speech advocates, often characterized by anti-vaccine, or anti-science attitudes), in line with our thematic analysis presented above, only a very small number of them (144 out of 10,967 accounts) exchanged tweets, hence demonstrating little overall engagement with each other (see Fig. 3: Internal conversational network (a), here anonymized).^9^

We then turned our attention to the ‘successful tweet network’ comprising those who contributed one or more tweets that passed the threshold for significant response, as defined above (*N* = 108). A closer analysis of the tweeters confirmed that the discussion was heavily US-led (apart from 9 users from Italy, 1 from Pakistan, 1 from India, 1 from Ireland, 1 from the UK, and 1 from Germany); 7 users described explicitly themselves as QAnon followers, 29 as MAGA/Trump supporters, and ten as (conservative) Christians; being part of traditional families, ‘pro-life,’ ‘free-thinkers,’ patriots, and NRA supporters were other recurrent themes in the users’ descriptions. Being ‘censored by Twitter’ in the past was mentioned as a badge of honor. In an attempt to find further similarities, we looked at who the 108 successful tweeters are following, revealing that the top most commonly followed accounts are realDonaldTrump (*N* = 74), POTUS (*N* = 57), WhiteHouse (*N* = 52), and FLOTUS (*N* = 51) (in line with the clustering evidenced by Sanders et al. 2021).

Being anti-European Union or anti-Euro, pro-Brexit or pro-Salvini (an Italian politician known for his right-wing populistic ideas) were recurring themes from the non-US-based accounts—confirming once more the variety of actors present under the #NoMask hashtags, and yet their being bonded by compatible narratives. This heterogeneity of ‘successful tweeters’ was to be found also by looking at their overall Twitter presence and popularity: some of the tweeters behind the most successful tweets joined Twitter more than a decade ago, some only over the last year; most of these tweeters did not have, in most cases, particularly high numbers of followers. Only three of them were beyond, or close to, 1-m followers. Most had a few thousand followers, 12 less than 1000, and 4 less than 100.

**Fig. 4:**
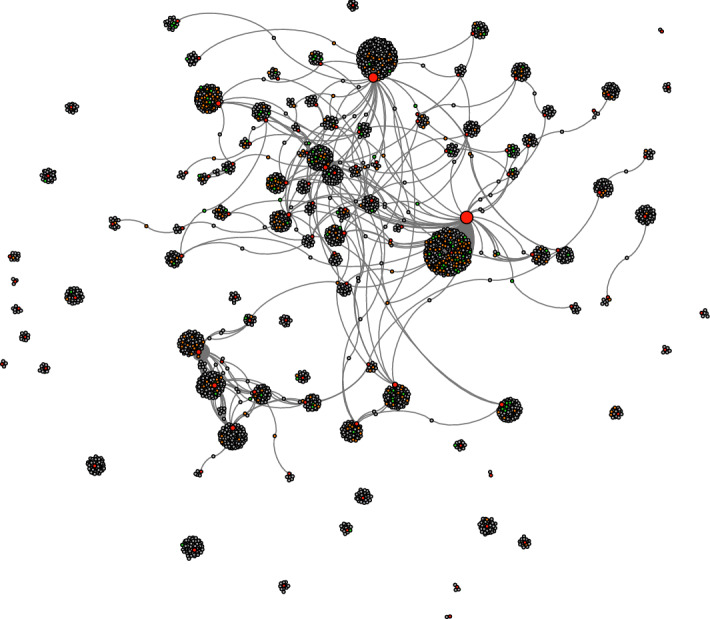
Responders to successful tweeters

Finally, we examined the responses to each of these successful tweets and looked at the accounts who wrote the response tweets in order to determine their ‘exogenous’ engagement—that is, whether the responses to the successful tweets came from the set of people who had used the hashtag in the first place or whether a completely different set of accounts was being engaged. We collected this additional data using the Web Data Research Assistant software, resulting in a total of 5889 responding tweets from 4866 unique accounts (please note that 8 of the 108 accounts that sent successful tweets have been suspended or deleted from the Twitter platform and their responses could not be collected). Figure 4 (Responders to successful tweeters) shows in graph form the responses between the users who posted successful tweets (shown in red) and the blob of accounts that responded (green if they had appeared in our complete network, orange if they appeared in the tweet network, and gray if they had not featured in the previous network at all). The vast majority of responding accounts have not been seen before (4386, or 90%) and 120 accounts (2.5%) had only been mentioned by tweets in our original dataset and had not themselves tweeted an anti-masks hashtag. 259 accounts (5%) that appeared in the responses to successful tweets had tweeted (non-successful) anti-mask tweets. The graph indicates that there is almost no overlap between the respondents and rather than a ‘community’ of respondents we have disconnected individuals engaging with the hashtags under observation—suggesting the broad reach of the successful tweets.

**Fig. 5:**
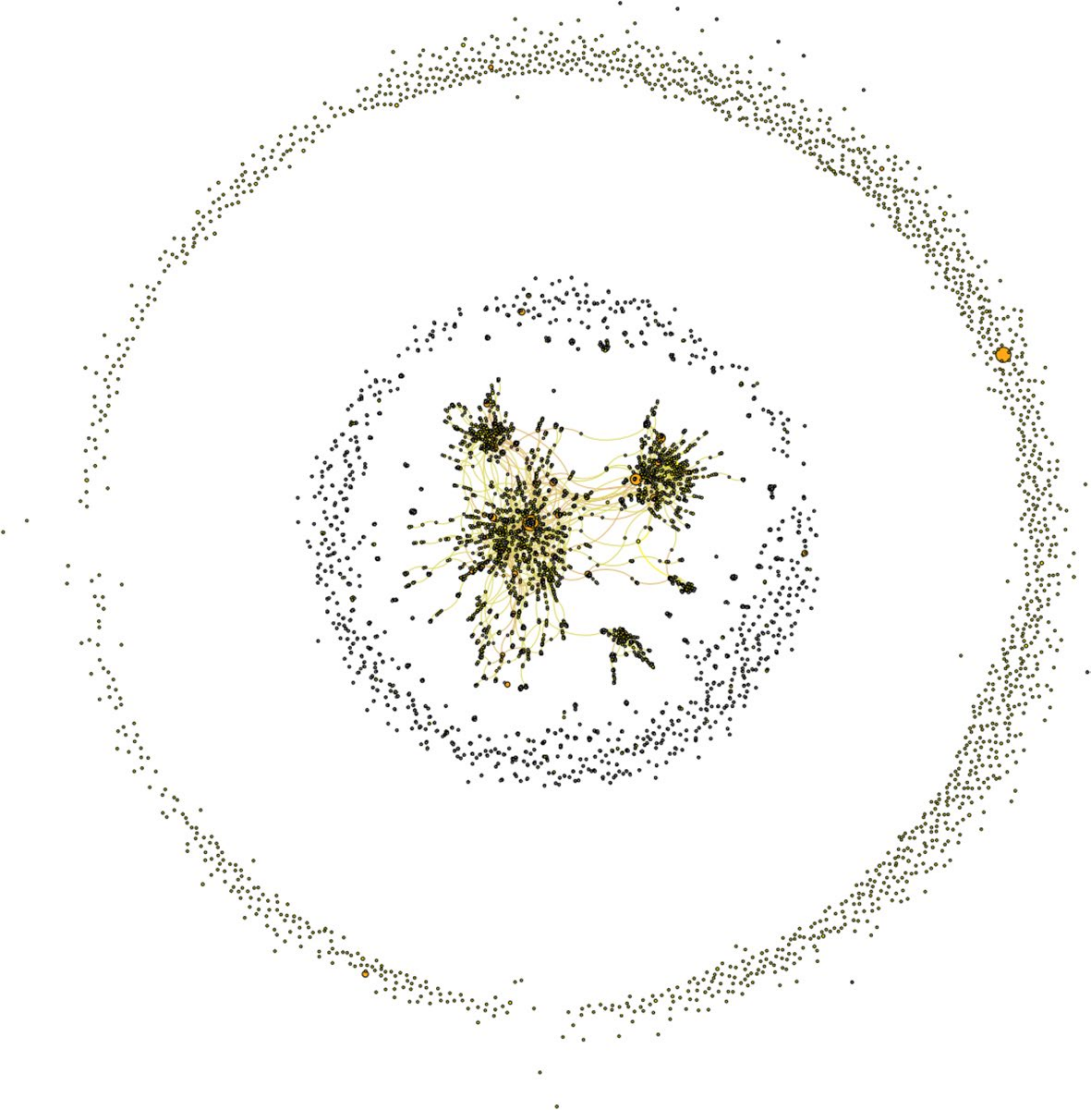
Complete mention network (b)

By carrying out similar network analyses with the more recent dataset, we were able to confirm similar patterns and to strengthen our interpretation of our results. First, the similarly ‘doughnut-shaped’ Complete mention networks (b) reveals at a first glance how poorly connected are most of the accounts considered and how for most users being part of the ‘NoMask’ digital conversation simply means an occasional engagement with the hashtags under consideration [Fig. 5].

Please note: the size and color of each node are related to the number of tweets that the account has contributed to our dataset, as in Fig. 2.

**Fig. 6:**
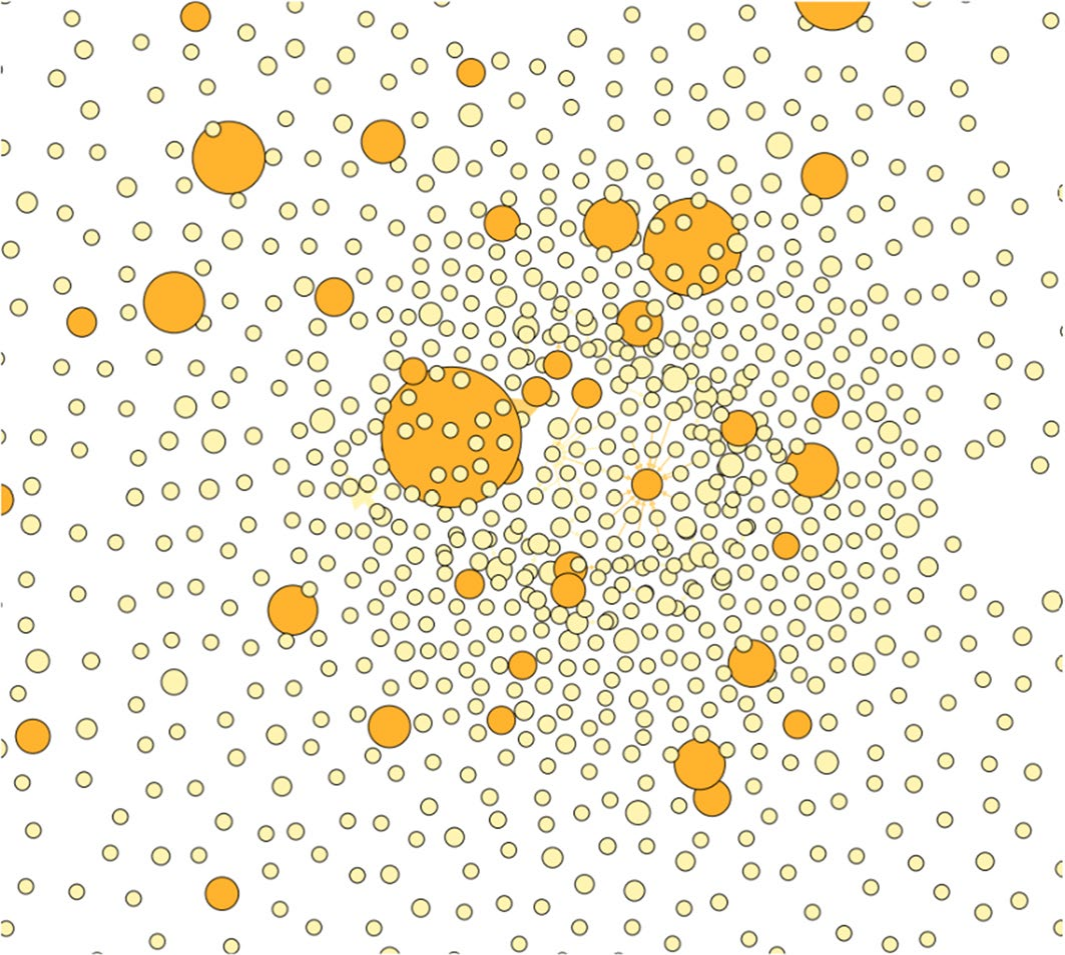
Basic tweet network (b)

Indeed, if we explore the basic tweet network (comprising all those who actively contributed one or more tweets in our dataset, hence excluding those who were only mentioned—see Fig. 6) and ‘zoom in’, the edges (i.e., the ‘links’) have disappeared: in other words, what was connecting the previous network up was the fact that the contributing tweet authors were all mentioning (or replying to) the same non-participating accounts.

**Fig. 7:**
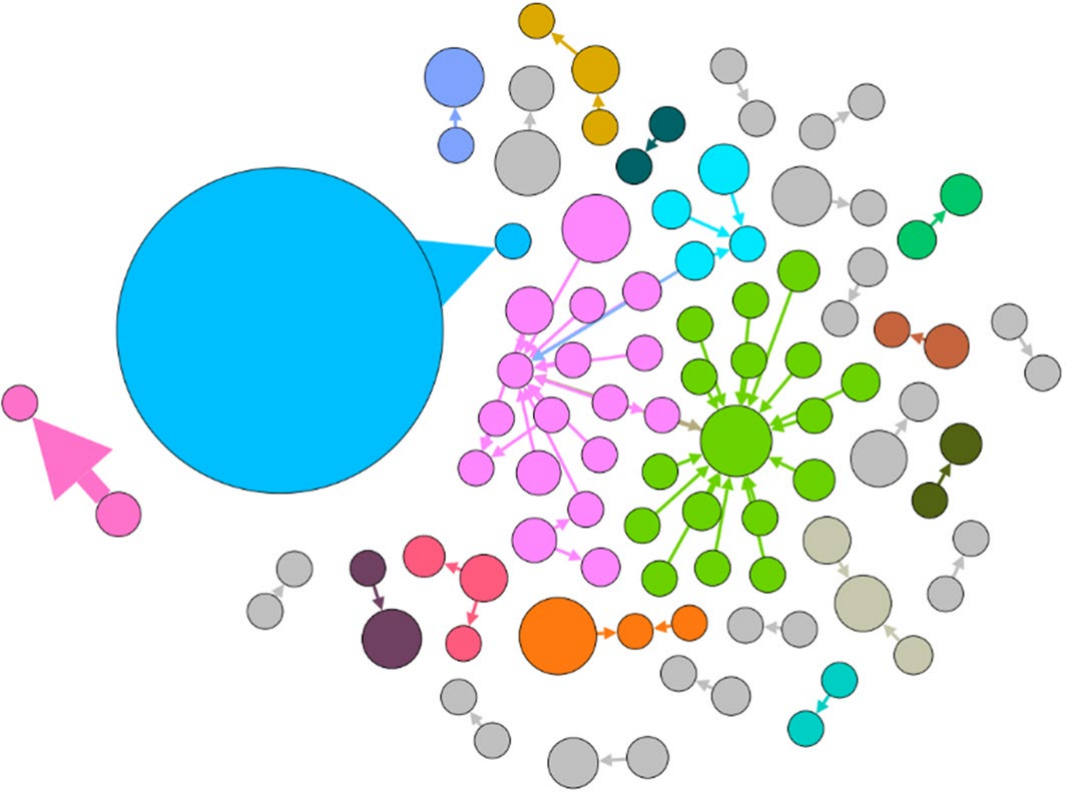
Internal conversational network (b)

The fact that also in the more recent dataset, despite promoting diverse but compatible discourses (as discussed in the thematic analysis), there was little engagement among users confirms the absence of a digital community, but only a thematic convergence of individuals and networks pivoting around different conversations and interests [e.g., see Fig. 7: Internal conversational network (b)].

## Further discussion and conclusions

This exploratory study analyzed the #NoMask discourses and conversations prevalent in the first and latest stages of the pandemic in the Twitter Anglosphere by bringing together network analyses and qualitative approaches. While we recognize that the methodological approach used has limitations when it comes to data generalizability, it allows us to draw some important conclusions. We have shown that, while there is not a #NoMask movement or community, we can identify a range of heterogeneous actors that, under NoMask-adjacent themes and hashtags, have been independently promoting diverse (but politically compatible and converging under the theme of ‘freedom’) discourses. The use of the hashtags analyzed by disparate online subcultures including anti-government extremists, anti-vaxxers, and QAnon adherents can contribute to the development of a more cohesive ideology; this in turn can expose mainstream audiences to more extreme ideologies, which can deepen and intensify beliefs and channel unfocused hatred toward political and cultural targets.

It is important to note that the lack of a structured network does not mean that algorithms do not have a key role as enablers of misinformation: they still shape the looser networks driving the convergent narratives that we observed. Indeed, in a social context in which classifications, predictions, and decisions (including decisions on information exposure) are frequently being made based upon algorithmic models (Leenes et al. 2017), the exposure of citizens to disinformation, fake news, and extremist propaganda has become a global socio-technical concern (European Commission 2018). We know, for instance, that extremists have increasingly utilized the technology offered by social media platforms in order to increase the volume and diversity of propaganda that is available, which has consequently altered the ways in which individuals are accessing and engaging with political information (DiFranzo and Gloria-Garcia 2017; Raymond 2019). As such, we believe that a socio-technical response is needed—one making the most of both sociological and computational expertise.

We have also confirmed that political drivers and conspiratorial thinking had a pivotal role in propagating potentially harmful (for both individual and public health) ideas related to the use of protective masks, fostering the conception that masks are a symbol for socio-political identification. From this perspective, public health communications or online activists insisting in underlying the preventive value of mask wearing (or, similarly, of vaccines and boosters) without trying to disentangling the identity construction value that protective health interventions now hold are set for failure. This is in line with the literature increasingly suggesting that for effective health-related communications the simple delivery of statistical information is not effective and in certain circumstances might rather be counterproductive, as many health-related decisions are driven by individual epistemologies and even emotionally driven decision-making (Fahlquist 2019; Duchsherer et al. 2020; Mede and Schafer 2020).

We have seen how the heterogeneity of the #NoMask people and the multiple variants of their discourses (for instance when in conjunction with QAnon, or with the ‘Freedom Convoy 2022’) represent a widespread mobilized digital conspiracy phenomenon linking health concerns and politics (see also Sanders et al. 2021) with other types of social harms. In this regard, our findings have important implications for other campaigns embedded in misinformation. As with other harmful online behaviors and activities—such as harassment tactics, crypto-financing, and the livestreaming of violence—security institutions, policy makers, and other stakeholders should expect the expansion and migration of mobilized digital conspiracies as a strategic practice going forward: by praying on people fears or vulnerabilities and exploiting converging narratives to expand potential audiences, digital conspiracies are increasingly becoming an insidious tool. Furthering psychological research on conspiratorial thinking and fake news (e.g., Greifender et al. 2020) and new studies (e.g., based on in-depth interviews or psychometric assessments with those engaged with potentially dangerous conspiratorial movements) are needed to examine the commonalities that may exist between conspiratorial belief and support for and active involvement in extremist violence or other types of radicalization, in order to facilitate the development of better (socio-technical) instruments for harm prevention and mitigation.

## Data Availability

The datasets generated and analyzed during the current study are not publicly available due to confidentiality issues, but are available on reasonable request by researchers and subject to ethical approval of request by the University of Southampton.

## Footnotes

1 This collection was timed to start just before the adoption of the #NoMask hashtag in the context of COVID-19; previously the hashtag had seen other use by the Hong Kong protests. Before starting with the automatic data collection, we manually checked for the oldest tweets using our selected hashtags with reference to the pandemic, and decided to start on 1st Feb 2020 as around that time we could start to observe a tweets relevant for our analyses. This is not surprising: on February 1 a WHO Situation Report came out detailing a number of cases from several countries (including the USA).

2 This second collection was timed to cover what have been considered the end stages of the pandemic, or at least of the last major ‘pandemic wave,’ in the countries that were clearly prevalent from the first data collection; at these time, pandemic restrictions were eased or were going to be eased soon, hence—we hypothesized—creating a potential change in the narratives used.

3 Available from http://southampton.ac.uk/~lac/WebDataResearchAssistant/, the software is a Chrome browser extension that monitors pages that the researcher browses (e.g., social media timelines and search results) and saves relevant data and metadata as a spreadsheet. This approach allows historic searches to be conducted (i.e., searches for data more than 10 days old), which mitigates well-known limitations of Twitter’s public API (e.g., issues of sampling and representativeness, lack of transparency, and reliability—as discussed in Lomborg and Bechmann 2014).

4 Using the open source python Twarc package created by Documenting the Now project (www.docnow.io), to obtain information not limited by the public API.

5 Most prominently the ‘Boogaloo’ movement and the American Militia Movement. The term Boogaloo is largely used online as a code word for Civil War. Adherents to this movement envision a conflict between armed citizens and law enforcement that ultimately results in the US government being violently overthrown (Argentino 2020). The American Militia Movement is part of the broader Patriot Movement. One of its most prominent characteristics is their support for the second amendment (‘The Right to Bear Arms’). For many Militia groups, the purpose of gun control ownership is the strong deterrent it serves against the infringement of power by would be tyrants and the protection it provides against despotism (Pitcavage 2001).

6 Originally, the Freedom Convoy (*Convoi de la liberté*) was about opposing the Canadian government’s vaccine mandates for truckers coming across the US border. By exploiting populist grievances, it evolved into a wider protest against vaccine mandates more generally, inflation, and Justin Trudeau’s liberal government. ‘The People’s Convoy’ is the US imitation that took place in Washington D.C. in similar protest against covid-19 restrictions.

7 That is, a series of protests and blockades in Canada against COVID-19 vaccine mandates and restrictions began in early 2022.

8 It is worth noting that the second data collection is subsequent to the events of January 6 2021, during which insurrectionists attempted to impeded Congress’s Constitutional mandate to validate the Presidential election and launched an assault on the United States Capitol Complex. This event led to a number of social media companies including Twitter removing Trump from its platform. As a consequence, also many of his followers left the platform, which explains we are likely seeing less pro-Trump support in the data.

9 Four accounts have particularly high betweenness centrality of the overall network, indicating their importance in allowing information to travel from one part of the network to another. Examination of each account’s tweets shows that they are highly invested in passing on information to other users and in sharing / using multiple hashtags and account mentions to attract attention.
